# What does a mouse tell us about *neuregulin 1*—cannabis interactions?

**DOI:** 10.3389/fncel.2013.00018

**Published:** 2013-02-26

**Authors:** Tim Karl, Jonathon C. Arnold

**Affiliations:** ^1^Neuroscience Research AustraliaRandwick, NSW, Australia; ^2^Schizophrenia Research InstituteDarlinghurst, NSW, Australia; ^3^School of Medical Sciences, University of New South WalesNSW, Australia; ^4^Department of Pharmacology, Bosch Institute, University of SydneyNSW, Australia; ^5^Brain and Mind Research InstituteCamperdown, NSW, Australia

**Keywords:** schizophrenia, cannabis, *neuregulin 1*, gene-environment interactions, mouse model

## Abstract

The link between cannabis and psychosis has been debated although there is substantial epidemiological evidence showing that cannabis increases the risk of psychosis. It has been hypothesized that schizophrenia patients carrying particular risk genes might be more sensitive to the psychosis-inducing effects of cannabis than other patients and healthy test subjects. Here we review the effects of cannabinoids on a mutant mouse model for the schizophrenia candidate gene *neuregulin 1* (*Nrg1*). The studies suggest a complex interaction between cannabis and *Nrg1*: the neuro-behavioral effects of cannabinoids were different in *Nrg1* mutant and control mice and depended on exposure time, sex, and age of test animals. This research provides the first evidence of complex cannabis-*Nrg1* interactions suggesting *Nrg1* as a prime target for future clinical investigations. Furthermore, it highlights that animal model research can broaden our understanding of the complex multi-factorial etiology of schizophrenia. Finally, the findings are important to preventive psychiatry: if the genes that confer genetic vulnerability to cannabis-induced psychosis were identified patients at-high risk could be forewarned of the potential dangers of cannabis abuse.

The two-hit hypothesis of schizophrenia states that a combination of genetic and environmental risk factors will cause the development of schizophrenia (Bayer et al., 1999; Rapoport et al., 2005; Caspi and Moffitt, 2006). Scores of genetic risk factors have been suggested for schizophrenia (Allen et al., 2008) and mouse mutants have been developed for most of those candidates (Desbonnet et al., 2009). *Neuregulin 1* (*NRG1*) is one of the more promising schizophrenia candidate genes as associations with schizophrenia have been found in several studies (Stefansson et al., 2002; Tosato et al., 2005; Munafo et al., 2006). However, similar to many other schizophrenia susceptibility genes, recent genome wide associations studies suggest that it is more important to consider an interplay of different genetic and environmental risk factors for schizophrenia to understand the etiology of the disorder (Sanders et al., 2008). Thus, many environmental factors have been considered as risk factors for schizophrenia (Van Os et al., 2010) and cannabis use has been the focus of an ever larger growing list of studies. Cannabis appears to be a component/cumulative cause for schizophrenia and increases the overall risk of developing the disorder by 2-fold (Henquet et al., 2005). Importantly, an increasing number of researchers believe that this risk might be elevated for cannabis users with a genetic vulnerability to schizophrenia (Caspi and Moffitt, 2006). Indeed, a functional polymorphism in the gene for catechol-*O*-methyl transferase (*COMT*) was implicated in conferring vulnerability to cannabis-induced psychosis (Caspi et al., 2005; Henquet et al., 2006). Subsequently, a genetic mouse model for *COMT* was treated with the main psychoactive component of cannabis (Δ^9^-tetrahydrocannabinol: THC) during adolescence and exhibited a greater behavioral sensitivity to the long-term effects of THC (O'Tuathaigh et al., 2010) and a genotype-specific response in dopaminergic and GABAergic pathways as well as in the protein expression of cannabinoid 1 receptors (CB1) (Behan et al., 2012). Based on *NRG1's* established role in schizophrenia and the availability of validated mouse mutants for *Nrg1* (Duffy et al., 2008), we investigated over the last decade if *Nrg1* represents a second candidate for gene-cannabis interactions in schizophrenia. The mini review will outline how our mouse research has been instrumental in discovering *Nrg1*-cannabis interactions relevant to schizophrenia and in deciphering potential mechanisms. Our studies not only considered THC but also the cannabinoid cannabidiol (CBD), which is devoid of psychoactive properties and has been reported to block or reverse effects of THC and have antipsychotic properties (Arnold et al., 2012).

The protein Nrg1 influences key neurodevelopmental processes such as myelination, synaptogenesis, neuronal migration, and is involved in the expression and activation of N-methyl-D-aspartic acid (NMDA) receptors (Harrison and Law, 2006; Mei and Xiong, 2008). Importantly, a number of genetic mouse models have been developed for the different isoforms of Nrg1 (Duffy et al., 2008; Mei and Xiong, 2008; Karl et al., 2011). Among those, the heterozygous transmembrane domain *Nrg1* mutant mouse (*Nrg1* HET) has shown compelling face, construct, and partial predictive validity for schizophrenia research (Stefansson et al., 2002; Karl et al., 2007, 2011; Van Den Buuse et al., 2009; Duffy et al., 2010; Chesworth et al., 2012a,b). Thus, our team has utilized this model to determine the nature of *Nrg1*-cannabis interactions in great detail (for review see Arnold et al., 2012). The clinical relevance of this research has recently been highlighted by a genome-wide linkage and single nucleotide polymorphism association analysis, which discovered *NRG1* as a major candidate for the development of cannabis dependence in African Americans (Han et al., 2012). The findings of our earlier mouse model research will be outlined in the following.

In an initial study we exposed *Nrg1* HET mice to acute doses of THC before testing them in an array of schizophrenia-relevant behavioral paradigms (Powell and Miyakawa, 2006). *Nrg1* mutant mice exhibited an increased sensitivity to the locomotor-suppressant and anxiogenic effects of THC compared to wild type-like littermates (WT). Surprisingly, the mutants also showed improved sensorimotor gating following THC challenge as measured by prepulse inhibition of the startle response (PPI) (Boucher et al., 2007a). Increased PPI is often detected after treatment with antipsychotic drugs which normalize PPI deficits of schizophrenia patients (Geyer et al., 2001). Recent human data suggest that *NRG1* may also confer increased behavioral sensitivity to THC [although *NRG1* polymorphisms worsened THC-induced information processing dysfunction rather than improving it (Stadelmann et al., 2010)]. It is possible that the effects of heterozygous deletion of *Nrg1* in mice might be opposite to that conferred by *NRG1* polymorphisms in patients. Future studies may examine whether mice overexpressing Nrg1 protein display exaggerated THC-induced PPI deficits. Follow-up experiments revealed that the enhanced behavioral response of *Nrg1* HETs to acute THC was sex-specific as female mutants showed no enhanced susceptibility to acute THC and actually developed resistance to aspects of THC-induced social withdrawal (Long et al., 2010a). It is unclear as to why *Nrg1*-cannabinoid interactions are sex-specific. Gender influences the actions of cannabinoids (McGregor and Arnold, 2007) and interactions between gonadal hormones and neuregulin have been demonstrated [(Lacroix-Fralish et al., 2006); but also see Taylor et al., 2011]. Future studies could examine whether Nrg1 expression regulates the modulatory effects of gonadal hormones on cannabinoid receptor sensitivity.

The increased behavioral susceptibility of male *Nrg1* HET mice to THC was accompanied by elevated neuronal activation as measured using c-Fos immunohistochemistry (Boucher et al., 2007b). THC selectively increased c-Fos expression in the ventral part of the lateral septum (LSV) of *Nrg1* mutants. No corresponding effect was observed in control littermates. Interestingly, drugs, which modulate PPI, whether they are pro-psychotic drugs that impair PPI, or anti-psychotic drugs that facilitate PPI, all increase c-Fos expression in the lateral septum (Sumner et al., 2004). Furthermore, *Nrg1* HET mice exhibited a more pronounced enhancement of c-Fos levels in stress-related brain regions (i.e., paraventricular nucleus of hypothalamus and central nucleus of amygdala). In summary, these animal studies provided the very first evidence for an interaction between the schizophrenia risk gene *Nrg1* and cannabis and implied that stress and gender may also influence these interactions.

Chronic cannabis use is more relevant in cannabis-induced psychosis than acute exposure. Thus, our team continued this line of research and determined the neuro-behavioral response of *Nrg1* mutants to long-term cannabinoid exposure. *Nrg1* mutant and control mice were treated chronically with the synthetic CB1 receptor agonist CP 55,940 (Boucher et al., 2011). *Nrg1* hypomorphic mice developed tolerance to the hypothermic and locomotor-suppressant effects of CP 55,940 more rapidly than WT mice. Interestingly, tolerance development toward the anxiogenic effects of the cannabinoid was only observed in control mice whereas *Nrg1* HETs maintained persistent THC-induced anxiety with repeated CP 55,940 dosing. All mice developed tolerance to the genotype-specific effects of acute CP 55,940 on PPI (i.e., impairment in WT and facilitation in *Nrg1* HET mice). Mutant mice showed a selective increase in CP 55,940-induced FosB/ΔFosB expression in the LSV, which is a marker for long-term neuroadaptive changes. These findings suggest that *Nrg1* is not only involved in the acute neuro-behavioral response to cannabinoids but also modulates neuroadaptive responses to long-term cannabinoid challenge. Furthermore, it confirms the LSV as an important brain region for *Nrg1*–cannabinoid interactions. This could be related to the fact that the lateral septum shares reciprocal projections with the hypothalamus and the amygdala and receives cognitive input from the hippocampus and the prefrontal cortex (Sheehan et al., 2004). These brain areas are important in schizophrenia and are characterized by high expression levels of Nrg1, its main receptor ErbB4 and CB1 (Law et al., 2004; Kofalvi, 2008; Neddens and Buonanno, 2011). Future studies should examine in more detail the role of the LSV in mediating the neuro-behavioral effects of cannabinoids in *Nrg1* HET mice and define in particular the involvement of CB1 and ErbB4 receptors.

Human research suggests that adolescence is a time of increased vulnerability to the detrimental effects of cannabis on the development of psychosis (Caspi et al., 2005). Thus, our team exposed adolescent WT and *Nrg1* HET mice to chronic THC (Long et al., 2013). Surprisingly, *Nrg1* mutants appeared less susceptible to THC-induced suppression of investigative social behaviors than control mice. However, adolescent THC exacerbated the hyperlocomotive phenotype characteristic for adult *Nrg1* mutant mice (Karl et al., 2007; Long et al., 2013). *Nrg1* deficiency also modulated the effects of adolescent THC on neurotransmitter systems involved in the pathophysiology of schizophrenia. Radioligand binding analyses found genotype-specific THC effects on CB1 expression in the substantia nigra: *Nrg1* HET mice exhibited reduced CB1 levels drug-free whereas CB1 binding was decreased in WT and increased in *Nrg1* mice post THC challenge. Lower CB1 expression levels in the substantia nigra might be responsible for the observed decreased susceptibility of adolescent *Nrg1* mutant mice (whereas binding studies in adult *Nrg1* HETs found increased levels of CB1 in the same brain region; manuscript currently being submitted). Interestingly, ErbB4 is localized in dopaminergic neurons in the substantia nigra (Abe et al., 2009). Thus, ErbB4 and CB1 might interact in the substantia nigra and thereby regulate the hyper-locomotor phenotype of *Nrg1* mutant mice. *Nrg1* also conferred opposing effects of THC on 5-HT2A receptor expression in the insular cortex and NMDA receptor binding was selectively increased in the hippocampus and cingulate cortex of *Nrg1* HET mice (Long et al., 2013) (for a better mechanistic understanding of *Nrg1*-THC interaction on NMDA receptor expression in the hippocampus see Spencer et al., 2013).

The *cannabis sativa* plant is a mix of over 60 different cannabinoids, one being THC, another being CBD, which blocks, or reverses the effects of THC and other psychotropic drugs such as methamphetamine (Long et al., 2010b). Varying levels of THC and CBD in different cannabis strains could modify the consequences of long-term cannabis consumption and also shift the nature of gene-cannabis interactions such as the one reported here for *Nrg1*. Thus, our team characterized the neuro-behavioral response of *Nrg1* HET mice to acute and chronic CBD (Long et al., 2012) to investigate its potentially therapeutic-like effects in animal models for schizophrenia. CBD did not alter schizophrenia-relevant behaviors such as hyperlocomotion or PPI deficits in our *Nrg1* HET mouse model (Long et al., 2012). Nevertheless, high dose CBD selectively increased social interaction of *Nrg1* mutant mice, which are normally characterized by diminished investigative social behaviors (i.e., social withdrawal) at baseline. Furthermore, chronic CBD also increased GABAA receptor binding in the granular retrosplenial cortex of mutant mice suggesting that *Nrg1* may not only modulate neuro-behavioral actions of THC but also of CBD in a task- and brain region-specific manner. Further research using a variety of CBD doses thereby considering dose and age-effects will address the issue of varying or even opposing effects of different cannabinoids more comprehensively.

In summary, the transmembrane domain *Nrg1* mouse model has enabled the detailed analysis of acute vs. chronic effects of cannabinoids at different stages of brain development. *Nrg1* modulated the behavioral sensitivity of mice to cannabinoids differentially during adolescence and adulthood providing evidence for a role of *Nrg1*-cannabis interactions in schizophrenia. Furthermore, insights into the molecular and neurobiological mechanisms of *Nrg1*-cannabinoid interactions (involvement of CB1, 5-HT2A and NMDA receptors in particular) would not have been possible without utilizing these mouse mutants. Future research will extend on our initial findings and address sex specificity and the opposite effects of CB1 stimulation in adolescence (*Nrg1* mutant less susceptible) and adulthood (*Nrg1* mutants more susceptible) in greater detail. Finally, models of cannabinoid addiction should be considered given the significant comorbidity of schizophrenia and drug dependence.

## Conflict of interest statement

The authors declare that the research was conducted in the absence of any commercial or financial relationships that could be construed as a potential conflict of interest.
